# Glycemic Disorder Risk Remote Monitoring Program in the COVID-19 Very Elderly Patients: Preliminary Results

**DOI:** 10.3389/fphys.2021.749731

**Published:** 2021-10-27

**Authors:** Abrar-Ahmad Zulfiqar, Delwende Noaga Damien Massimbo, Mohamed Hajjam, Bernard Gény, Samy Talha, Jawad Hajjam, Sylvie Ervé, Amir Hajjam El Hassani, Emmanuel Andrès

**Affiliations:** ^1^Service de Médecine Interne, Diabète et Maladies Métaboliques de la Clinique Médicale B, Hôpitaux Universitaires de Strasbourg et Equipe EA 3072 “Mitochondrie, Stress Oxydant et Protection Musculaire,” Faculté de Médecine-Université de Strasbourg, Strasbourg, France; ^2^Predimed Technology Society, Schiltigheim, France; ^3^Faculté de Médecine-Université de Strasbourg, Service de Physiologie et d’Explorations Fonctionnelles, Hôpitaux Universitaires de Strasbourg et Equipe EA 3072 “Mitochondrie, Stress Oxydant et Protection Musculaire,” Strasbourg, France; ^4^Centre d’Expertise des TIC pour l’Autonomie (CenTich) et Mutualité Française Anjou-Mayenne (MFAM)-Angers, Angers, France; ^5^Laboratoire IRTES-SeT, Université de Technologie de Belfort-Montbéliard (UTBM), Belfort, France

**Keywords:** diabetes risk, COVID-19, remote monitoring, *MyPredi*^TM^ platform, GER-e-TEC study, prevention, elderly patients

## Abstract

**Introduction:** The coronavirus disease 2019 (COVID-19) pandemic has necessitated the use of new technologies and new processes to care for hospitalized patients, including diabetes patients. This was the basis for the “GER-e-TEC COVID study,” an experiment involving the use of the smart *MyPredi*^TM^ e-platform to automatically detect the exacerbation of glycemic disorder risk in COVID-19 older diabetic patients.

**Methods:** The *MyPredi*^TM^ platform is connected to a medical analysis system that receives physiological data from medical sensors in real time and analyzes this data to generate (when necessary) alerts. An experiment was conducted between December 14th, 2020 and February 25th, 2021 to test this alert system. During this time, the platform was used on COVID-19 patients being monitored in an internal medicine COVID-19 unit at the University Hospital of Strasbourg. The alerts were compiled and analyzed in terms of sensitivity, specificity, positive and negative predictive values with respect to clinical data.

**Results:** 10 older diabetic COVID-19 patients in total were monitored remotely, six of whom were male. The mean age of the patients was 84.1 years. The patients used the telemedicine solution for an average of 14.5 days. 142 alerts were emitted for the glycemic disorder risk indicating hyperglycemia, with an average of 20.3 alerts per patient and a standard deviation of 26.6. In our study, we did not note any hypoglycemia, so the system emitted any alerts. For the sensitivity of alerts emitted, the results were extremely satisfactory, and also in terms of positive and negative predictive values. In terms of survival analysis, the number of alerts and gender played no role in the length of the hospital stay, regardless of the reason for the hospitalization (COVID-19 management).

**Conclusion:** This work is a pilot study with preliminary results. To date, relatively few projects and trials in diabetic patients have been run within the “telemedicine 2.0” setting, particularly using AI, ICT and the Web 2.0 in the era of COVID-19 disease.

## Introduction

Intensive glucose control has been shown to delay or prevent the development of micro- and macro-vascular complications related to diabetes (Andrès et al., 2018a).

In this context, telemedicine may be an effective approach to solve problems of education, compliance, and monitoring and provider access (Andrès et al., 2018a). Thus, telemedicine is likely to provide assistance and even optimize the management of these chronic conditions, in particular by avoiding certain emergencies and repeated hospitalizations, as demonstrated in the field of heart failure and diabetes through the use of remote monitoring or remote follow-up (Chaudhry et al., 2010; Anker et al., 2011; Andrès et al., 2018c; Seferovic et al., 2019; Zulfiqar et al., 2019).

Since the 2010–2015, more numerous mature telemedicine projects and studies have been developed in the setting of diabetes management, especially in the setting of telemonitoring like The Utah Remote Monitoring Project (Shane-McWhorter et al., 2014), or the DIABETe project which was developed to optimize home monitoring of diabetic patients *via* a telemonitoring 2.0 platform, situations with a risk of decompensation of diabetes and its cardiovascular complications (Andrès et al., 2018a). Over the last decade, the Internet has become increasingly popular and is now an important part of our daily life. The use of “Web 2.0” technologies in health/medicine care or in telemedicine is referred to as “Health 2.0” or “Medicine 2.0,” and “telemedicine 2.0.”

To date, relatively few projects and trials in diabetic patients have been run within the “telemedicine 2.0” setting, particularly using AI (Artificial Intelligence), ICT (Information and Communication Technologies) and the Web 2.0. The new generation telemedicine projects in diabetes (Telemonitoring and Health Counseling for Self-Management Support from Lindberg et al. (2017), TELESAGE (Charpentier et al., 2011), DIABETe) are often known as “telemedicine 2.0” projects, given that they all utilize new Information and Communication Technologies (ICT) and the Web (tools for the “e-Health 2.0”).

The COVID-19 epidemic, which has been raging in France since February 2020, is strongly impacting the Alsace region and patients with severe cases of COVID-19 need to be hospitalized.

These severe cases of COVID-19 are the reason for prolonged hospital stays and high morbidity and mortality rates. In addition, many complications can occur, directly related to the disease due to SARS-CoV-2 (secondary immunodepression and opportunistic infections, glycemic decompensation, chronic respiratory failure, chronic heart failure, stroke, chronic kidney failure, etc.).

The current understanding and the epidemiological evolution of the disease suggest that the number of chronic pathologies is increasing, and that therefore, it is essential to optimize the screening and management of the chronic complications of COVID-19.

The coronavirus disease 2019 (COVID-19) pandemic has necessitated the use of new technologies and new processes to care for hospitalized patients, including diabetes patients. Diabetes is strongly associated with a poor prognosis in COVID-19. Scrupulous blood sugar management is crucial, since the worse outcomes are closely associated with higher blood sugar levels in COVID-19 infection (Pranata et al., 2021).

Our team developed a remote monitoring platform designed to help prevent the deterioration of geriatric syndromes: *GER-e-TEC* project (Zulfiqar et al., 2020a, b).

The main goal of *GER-e-TEC* study is to evaluate the use of a remote monitoring platform as a means of structuring and standardizing the medical care of dependent elderly patients to prevent glycemic disorder risk. Below, we present an illustration of the use of the *MyPredi*^TM^ platform for the glycemic remote monitoring in COVID-19 older diabetic patients.

## Patients and Methods

### Objective

The central objective of the present phase of the *GER-e-TEC* COVID pilot study is to evaluate the use of a remote monitoring platform, here the *MyPredi*^TM^ platform (Zulfiqar et al., 2020a), and to experiment with this telemonitoring solution for diabetic elderly patients affected by the SARS-CoV-2 virus (COVID-19) to prevent glycemic disorder risk. Our project has been developed to optimize hospital monitoring of diabetic elderly patients affected by COVID-19, *via* a telemonitoring 2.0 platform.

The AI of the remote monitoring platform (MyPredi platform) automatically generates indicators of “health status” deterioration, i.e., “warning alerts” for any chronic disease worsening, particularly diabetes, its macrovascular complications and cardiovascular comorbidities (e.g., arterial hypertension, chronic heart failure).

### Patients

This study took place during the 3rd wave of the epidemic in France, during the period extending between December 14th, 2020 and February 25th, 2021, conducted in the Department of Internal Medicine, Diabetes, and Metabolic Disorders at the University Hospital of Strasbourg (HUS, Strasbourg, France). Any diabetic patient over 65 years of age with COVID-19 infection admitted to the hospital or emergency room with one or more chronic diseases was eligible for the *GER-e-TEC* study. All patients with confirmed COVID-19 identified by positive results on reverse transcription polymerase chain reaction (RT-PCR) of nasopharyngeal swabs were included (SARS-CoV-2–Gene RdRp).

Patients with COVID-19-related infection who were monitored by the telemedicine solution for less than 48 h were excluded. Minors, pregnant women, patients who were unable to sign the eligibility and consent form, elderly patients in palliative care (i.e., patients in hospice with life-limiting diseases), and patients who refused to provide their consent were excluded from the study. If patients presented signs of severe dementia, the consent of their legal guardian was required.

### Study Outline

During the experiment, the patients recorded their vital signs every day with the help of smart devices. This data was then sent directly to the intelligent platform to be processed and analyzed in the unit. The platform uses an algorithm to anticipate risk situations for diabetic patients.

During the experiment, the alerts were compiled in the order they were received. They were analyzed with regard to the clinical context at the time they were emitted, using the discharge letter and computer files (medical and nursing) of the patient in question. This analysis was performed retrospectively by two professionals involved in the present study in the unit but not in contact with the caregivers who cared for the patients on a daily basis. The alerts were classified as “pertinent” or “non-pertinent” i.e., whether or not they were associated with an action or intervention by the clinic.

For the glycemic disorder risk, the threshold for triggering an alert for hypoglycemia was set at 0.7 g/L; for hyperglycemia, the threshold was set at 2 g/L; this in accordance with current recommendations.

### Experimental Protocol

**FIGURE 1:**
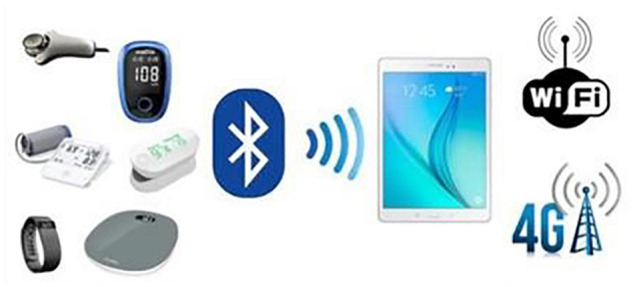
Ger-e-Tec’s connected non-intrusive medical sensors. Copyright: Predimed Technology, Schiltigheim, France.

The *MyPredi*^TM^ solution (Zulfiqar et al., 2020a, b) (i.e., a tablet and connected sensors) was used to collect the patient’s physiological data three times per day (morning, noon, night) and whenever the nurses and doctors checked in on the patient (3 and 2 times per day, respectively). A number of physiological measurements were taken, including blood pressure, heart rate, weight, oxygen saturation, capillary blood glucose, temperature, physical activity, and sleep. Questionnaires were also used [daily stool frequency; Confusion Assessment Method (CAM) questionnaire; pain questionnaire; biological measurements and iatrogenesis questionnaire (Zulfiqar et al., 2020b)]. See Figure 1 the sensors.

### The Remote Monitoring Platform

The *MyPredi*^TM^ platform is a generic platform with an original architecture and proven capabilities to refer patients with chronic pathologies who require long-term management.

This remote monitoring platform is based on non-intrusive medical sensors, allowing the collection of capillary blood glucose, blood pressure, heart rate, arterial oxygen saturation (SaO2), temperature and body weight. These sensors communicate *via* Bluetooth, enabling real-time feedback of physiological information on the patient’s health status. The platform also includes a touch tablet, which communicates *via* Wi-Fi with a box, or *via* 3G/4G, enabling interaction with the patient and providing nutritional-hygienic and therapeutic education. The *MyPredi*^TM^ system includes a server that hosts patient data and a secure Internet portal (website), allowing the patient and the various healthcare professionals to connect.

*MyPredi*^TM^ is based on an “intelligent” system in the form of an inference engine and a medical ontology, enabling personalized data analysis, which is specific to each patient, in real time or delayed mode, with, ultimately, the generation of “alerts.” The *MyPredi*^TM^ platform generates “indicators of deterioration in the patient’s health status,” known as “alerts,” in relation to a decompensation of chronic pathologies, in this case diabetes, with hyperglycemia and hypoglycemia alerts. This reasoning is based on an inference engine whose rules are created by medical experts (here, diabetologists, internists, geriatricians). The medical knowledge is derived from “evidence-based medicine.”

For the glycemic disorder risk, the threshold for triggering an alert for hypoglycemia was set at 0.7 g/L; for hyperglycemia, the threshold was set at 2 g/L; this in accordance with current recommendations.

For hypoglycemia, an alert was considered critical when capillary blood glucose was below 0.5 g/L; for hyperglycemia, an alert issued was considered mild when capillary blood glucose was between 2 and 2.5 g/l, moderate when capillary blood glucose was between 2.5 and 3 g/L and critical above 3 g/L.

### Parameters Evaluated and Statistical Analyses

We calculated sensitivity, specificity, and positive and negative predictive value for alerts issued for the geriatric risk, in order to determine the clinical relevance of the alerts generated by the *MyPredi*^TM^ remote monitoring platform for the glycemic disorder risk. Survival analyses were estimated using the Kaplan–Meier method. For the comparison of living and deceased elderly patients, we used the Student *t*-test and the Wilcoxon test.

We used *RStudio* software^1^ and *R code* (Boston, United States).

### Administrative Requirements

All the COVID-19 older diabetic patients who participated in the *GER-e-TEC* project were required to sign a consent form. A clinical research protocol for the *GER-e-TEC* project was filed with the *Ethics Committee of the Faculty of Medicine of Strasbourg* under the number RNI 2020–HUS N°7792. We also obtained authorization to conduct the study from the *Commission Nationale Informatique et Liberté* (CNIL, “National Commission on Informatics and Liberty”).

## Results

### Characteristics of Patients

A total of 71 patients were hospitalized in the internal medicine unit between December 14th, 2020 and February 25th, 2021. Of these, 10 diabetic elderly patients affected by COVID-19 disease were monitored remotely, while there was no refusal.

The mean age of the patients was 84.1 years with a standard deviation of 6.9 years. The median age was 84.2 years. There were 6 (60%) male patients and 4 female patients: a male/female ratio of 1.5 to 1. The mean HbA1c was 7.52% (Anker et al., 2011; Charpentier et al., 2011; Shane-McWhorter et al., 2014; Lindberg et al., 2017; Zulfiqar et al., 2020a; Pranata et al., 2021).

The patients used the telemedicine solution for an average of 14.5 days with a standard deviation of 9.9 days.

Among the 10 diabetic patients, 7 (70%) had a history of hypertension, 4 (40%) had a history of dyslipidemia, and 4 (40%) had a history of cognitive disorders. See Table 1 for more information on the accompanying syndromes. The average number of drug treatments at the time of admission was 8.5 with a standard deviation of 4.7. See Table 1 for more information on the treatments. The mean Charlson score, that predicts the 1-year mortality, was 7.1 with a standard deviation of 1.

**TABLE 1:** Characteristics of the study population (*n* = 10).

**Medical characteristics (*n*, %)**	
**Medical history**	
Arterial hypertension	7 (70%)
Atrial fibrillation	3 (30%)
Coronary syndrome	1 (10%)
Obliterating arteriopathy of the lower limbs	1 (10%)
Sleep apnea syndrome	1 (10%)
Phlebitis/pulmonary embolism	2 (20%)
Dyslipidemia	4 (40%)
Chronic renal deficiency	1 (10%)
COPD^*^	1 (10%)
Solids neoplasms	2 (20%)
Peptic ulcer	1 (10%)
Hypothyroidism	1 (10%)
Connectivities	1 (10%)
Cognitive disorder	4 (40%)
Obesity	2 (20%)
**Treatment**	
Beta blockers	5 (50%)
ACE inhibitors, Sartan	5 (50%)
Diuretics	3 (30%)
Calcium channel blockers	3 (30%)
Urapidil	2 (20%)
Anticoagulants	2 (20%)
Antiplatelet agents	2 (20%)
Statins	3 (30%)
Biguanide	4 (40%)
Sitagliptin	1 (10%)
Gliclazide	2 (20%)
Insulin therapy	2 (20%)
Benzodiazepines	3 (30%)
Antipsychotics	1 (10%)
Antidepressant	1 (10%)
Proton pump inhibitors	4 (40%)
L-Thyroxin	2 (20%)
Antiarrhythmics	2 (20%)
Pregabalin	3 (30%)
Morphin	1 (10%)
Amitriptyline	1 (10%)
**Symptoms at onset of illness COVID-19**	
Fatigue	4 (40%)
Confusion	2 (20%)
Dyspnea	8 (80%)
Fever	4 (40%)
Cough	3 (30%)
Diarrhea	1 (10%)
Acute heart failure	1 (10%)
Pulmonary embolism/phlebitis	2 (20%)
**Total lung involvement Chest CT Findings in Coronavirus Disease-19 (COVID-19)**	
Minimal (<25%)	7 (70%)
Moderate (25–50%)	1 (10%)
Severe to critical (≥75%)	2 (20%)

*COPD, chronic obstructive pulmonary disease.

All of patients had positive result for SARS-CoV-2 confirmed by reverse transcriptase PCR (RT-PCR) tests on nasopharyngeal swabs (thereafter referred to as COVID-19 diabetic patients).

The average length of stay was 13.6 days (4–30).

Of the 10 diabetic COVID-19 patients, 7 (70%) lived at home and 3 (30%) lived in nursing homes. After hospitalization, 5 (50%) patients returned to their homes and 5 (50%) died during the experiment.

### Data From the Sensors/Questionnaires

The MyPredi^TM^ remote monitoring solution collected a total of 44,571 measurements while monitoring the geriatric syndromes of the entire patient group. On average, 4,457 measurements were recorded per patient for geriatric disorders and risks. On average, 312 measurements were recorded per patient per day.

Mean blood pressure was 100.75 mmHg with a standard deviation of 9.11. The mean heart rate was 81.2 bpm with a standard deviation of 12.8 bpm. Mean oxygen saturation was 93.8% with a standard deviation of 4.9. The mean blood glucose level was 176.5 mg/L, with a standard deviation of 87.4 mg/L.

Mean activity was 1,027.1 steps per day with a standard deviation of 1,508.6 steps per day. The mean daily activity index was 13.6%. Mean sleep per day was 493.2 min with a standard deviation of 139.5 min. Mean light sleep was 162.5 min with a standard deviation of 118.2 min. Mean deep sleep was 330.8 min with a standard deviation of 144.6 min. The mean VAS (visual analog scale) was 0.1. The mean EVS (simple verbal scale) was 0.0. The mean Algoplus was 1.5 with a standard deviation of 1.8. The mean weight was 76.7 kg with a standard deviation of 21.7 kg. The mean number of stools was 0.3 stools per day with a standard deviation of 0.6. The mean temperature was 36.5°C with a standard deviation of 0.9°C.

The mean albumin was 36.2 g/L, with a standard deviation of 4.6 g/L; the mean natremia was 140.9 mmol/L, with a standard deviation of 5.4 mmol/L; the mean kalemia was 4.2 mEq/L, with a standard deviation of 0.6 mEq/L, and the mean creatinine was 90.8 μmol/L, with a standard deviation of 28.6 μmol/L.

### Number of Alerts for Diabetes Risk

During the monitoring, 142 alerts were emitted for the “glycemic disorder” risk indicating hyperglycemia, with an average of 20.3 alerts per patient and a standard deviation of 26.6; with 69 of these alerts classified as “mild,” 38 classified as “medium,” and 35 classified as “critical.”

In our pilot study, we did not note any hypoglycemia so the system emitted no alerts.

### Clinical Relevance of Alerts

Table 2 illustrates the clinical relevance of the alerts in terms of Se, Spe, PPV, and NPV for the evaluated criteria. Note the sensitivity of 100% for the alerts and the high negative predictive value.

**TABLE 2:** Sensitivity (Se), specificity (Spe), positive and negative predictive values (PPV; NPV) for alerts from the *MyPredi*^TM^ remote monitoring platform.

	**Glycemic disorder risk**
Sensitivity	100%
Specificity	–
Positive predictive value	100%
Negative predictive value	–

### Survival Analyses

**FIGURE 2:**
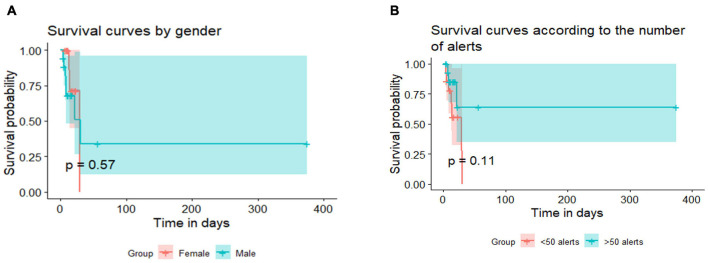
Survival analysis. **(A)** Survival curves by gender. **(B)** Survival curves by number of alerts. Copyright: Predimed Technology, Schiltigheim, France.

Survival analyses showed that gender played no role in the length of the hospital stay (a), regardless of the reason for the hospitalization (COVID-19 management). The analyses revealed that the length of the hospital stay was not affected also by the number of alerts in COVID-19 diabetic older patients (b). See results Figure 2.

### Clinical Relevance of Alerts for the Other Geriatric Risk

Table 3 indicates alerts and recommendations issued for the other geriatric risks studied in the 10 diabetic elderly COVID patients. Risks associated with and iatrogenesis (*n* = 175), decompensated heart failure (*n* = 126), and hypertension (*n* = 90) generated the most alerts.

**TABLE 3:** Alerts and recommendations issued for the other geriatric risks studied in the 10 diabetic elderly COVID patients.

	**Alert emitted *n***	**Recommendations *n***
Risk related to iatrogenesis (+)	175	0
No risk related to iatrogenesis (−)	112	106
Risk of bed rest (+)	52	0
No risk of bed rest (−)	0	0
Risk of constipation (+)	8	0
No risk of constipation (−)	19	19
Risk of decompensated heart failure (+)	126	0
No risk of decompensated heart failure (−)	0	0
Risk of pain (+)	0	0
No risk of pain (−)	0	0
Risk of dehydration (+)	11	0
No risk of dehydration (−)	10	10
Risk of alteration in heart rate (+)	1	0
No risk of alteration in heart rate (−)	0	0
Risk of malnutrition (+)	8	0
No risk of malnutrition (−)	15	15
Risk of confusion (+)	8	0
No risk of confusion (−)	0	0
Risk of fever (+)	7	0
No risk of fever (−)	0	0
Risk of hypo- and hyperkalemia (+)	6	0
No risk of hypo- and hyperkalemia (−)	14	14
Risk of hypo- and hypernatremia (+)	9	0
No risk of hypo- and hypernatremia (−)	10	10
Risk of hypertension (+)	90	0
No risk of hypertension (−)	0	0

For all geriatric risks, there were an average of 17 alerts per patient; we had, on average, 4.6 alerts per day and per patient with a median of three alerts per day, per patient.

There were on average five recommendations per patient for all geriatric risks, with a standard deviation of 6.8. We had, on average, two recommendations per day, per patient and per geriatric risk.

### Sensitivity, Specificity, Positive Predictive Values and Negative Predictive Values of Alerts for Other Geriatric Risks From the MyPredi Telemonitoring Platform for the 10 Elderly Diabetics Affected by COVID-19

Table 4 illustrates the clinical relevance of the alerts in terms of Se, Spe, PPV, and NPV for the other geriatric risk. Note the sensitivity of 100% for the alerts of all the other evaluated geriatric risks and the high negative predictive value.

**TABLE 4:** Sensitivity, specificity, positive and negative predictive values for alerts from the MyPredi^TM^ remote monitoring platform.

	**Decompensated heart failure**	**Pain**	**Dehydration**	**Brady- and tachycardia**	**Constipation**	**Bed rest**	**Malnutrition**	**Iatrogenia**	**Confusion**
Sensitivity	100%	–	100%	100%	100%	100%	100%	100%	100%
Specificity	–	–	50%	–	50%	–	50%	49%	–
Positive predictive value	100%	–	52%	100%	30%	100%	35%	61%	100%
Negative predictive value	–	–	100%	–	100%	–	100%	100%	–

	**Fever**		**Hypo- and hyperkalemia**			**Hypo- and hypernatremia**			**Hypertension**

Sensitivity	100%		100%			100%			100%
Specificity	–		50%			50%			–
Positive predictive value	100%		30%			47%			100%
Negative predictive value	–		100%			100%			–

### Elderly Diabetic Patients COVID-19 Alive vs Elderly Diabetic Patients COVID-19 Deceased

We present the results of the comparison of elderly patients who survived COVID-19 infection to the series of elderly patients who died from COVID-19 infection. We note heart rate, hyperglycemia, and the amount of deep sleep are significantly higher in elderly diabetics who died from COVID-19 infection. In addition, oxygen saturation, physical activity and the daily frequency of stools are significantly lower in elderly diabetics who died from infection linked to COVID-19.

See Table 5 for more results.

**TABLE 5:** Elderly patients Covid-19 alive VS elderly patients Covid-19 deceased.

**General data**	**Elderly diabetic patients Covid-19 alive (*n* = 5)**	**Elderly diabetic patients Covid-19 deceased (*n* = 5)**	***p***
Age	84.5 (±1.2)	83.9 (±9.3)	0.619
Average use of the telemedicine solution (days)	21 (±11.2)	10.2 (±6.6)	0.9257
Average number of drug treatments	8.2 (±7.3)	8.7 (±2.9)	0.5405
Charlson score	6.8 (±0.5)	7.3 (±1.2)	0.9443
Average measurements recorded per patient for geriatric disorders	8,021	2,752	0.08333
Average measurements recorded per patient per day	334	290	0.9659
Albumin level	38.8 (±7.4)	34.9 (±2.3)	0.8125
Natremia	138.2 mmol/L (2.8 mmol/L)	143.2 mmol/L (6.1 mmol/L)	0.000438
Kalemia	4.5 mEq/L (±0.4 mEq/L)	4 mEq/L (±0.7 mEq/L)	0.0007039
Creatinine level	102.7 μmol/L (20.6 μmol/L) 90.8 μmol/L (28.6 μmol/L)	0.9067	
Stool frequency	0.4 stools per day (±0.6)	0.1 stools per day (±0.5)	6.722e-05
Arterial pressure	105.71 mm Hg (±3.9 mm Hg)	100.77 mm Hg (±9.13 mm Hg)	0.8573/0.9988
Heart rate	76.9 bpm (±9.9 bpm)	84.7 bpm (±15.7 bpm)	0.0002023
Oxygen saturation	95.2% (±2.4)	91.2% (±6.2)	4.049e-08
Blood glucose level	167.4 mg/L (±97.2 mg/L)	212.1 mg/L (±59.8 mg/L)	9.409e-10
Weight	94 kg (± 26.8 kg)	70.3 kg (± 13.5 kg)	0.05146
Temperature	36.6°C (±0.7°C)	36.5°C (±1.1°C)	0.8196
Physical activity (median)	658 steps per day	93 steps per day	4.585e-06
Daily activity index	19.8% (±14.9%)	5.4% (±5%)	0.0001734
VAS pain score	0.1 (±0.4)	0 (±0.2)	0.5947
VRS pain score	0.1 (±0.2)	0 (±0.2)	0.7139
Amount of sleep	489 min per day (±112.6 min)	491 min per day (±156.9 min)	0.1773
Amount of light sleep	185.5 min per day (±127.1 min)	125.8 min per day (±91.6 min)	0.991
Amount of deep sleep	303.5 min per day (±149.8 min)	365.3 min per day (±133.9 min)	0.01704
Vitamin D	12.1 ng/ml (± 5.5)	22.9 ng/ml (±18)	0.7262

## Discussion

Glucose monitoring plays a crucial role in glucose control, which has been shown to be significantly related to the mortality risk of the new coronavirus disease 2019 (COVID-19) (Zhu et al., 2020). Elders, especially those with diabetes, are at the highest risk of COVID-19 related adverse outcomes and mortality. The COVID-19 pandemic has necessitated the use of new technologies and new processes to care for hospitalized patients, including diabetes patients. In the literature, some projects developed the use of remote real-time continuous glucose monitoring (CGM) in hospital like Davis et al. (2021), in a COVID-19 intensive care unit with nine patients included.

In our pilot study, COVID-19 infection clearly impacted the experiment we conducted, five elderly diabetic people with COVID-19 died. In our pilot study beyond hemodynamic parameters, such as oxygen saturation being significantly lower in elderly people with COVID who died, and hyperglycemia being significantly higher in elderly people with COVID who died, we noted that physical activity measured by the pedometer and the frequency of stools were significantly lower in diabetic elderly people with COVID-19 infection who died.

Unlike our work, the vast majority of telemedicine projects concerning diabetes have concerned the management or care of diabetic subjects during the period of the pandemic. We did not find any work on the glycemic monitoring of diabetic subjects affected by COVID-19 on the main scientific search engines (PubMed, Google Scholar).

To our knowledge, this is the first experimentation carried out with elderly patients suffering from COVID and who have multiple pathologies, with a telemedicine solution enabling the upstream detection of situations likely to degenerate into hyperglycemia or hypoglycemia. To our knowledge, this is one of the first projects that uses AI in addition to ICT (Information and Communication Technologies), which also evaluates other geriatric risk like the physical activity, constipation, dehydration, confusion and iatrogenesis.

At this level, we are in the context of predictive medicine, which is also personalized in the context of the *MyPredi*^TM^ platform, i.e., adapted to the phenotype of each older patient. On the other hand, it seems that this is the first time that such a communicative and “intelligent” system has been developed with the support of new technology tools, foreshadowing telemedicine 2.0 solutions.

All patients and health professionals used the *MyPredi*^TM^ system without any problems during the entire experiment. Thus, in our experience, age does not appear to be a limiting factor in the adoption and use of new technologies. Several recent studies have pointed in the same direction, documenting the use of telemedicine solutions, including among octogenarians. The telemonitoring platform used in our pilot study was first validated in a monocentric study conducted in the same hospital, primarily focused on the problem of heart failure (Andrès et al., 2015, 2018b, 2019a, 2019b). One hundred and seventy-five patients (mean age of 72 years) were included, 30% of the patients suffered from type 2 diabetes. During this first study, 1,500 measurements were taken to generate 700 alerts in 68 patients. One hundred and seven subjects (61.1%) had no alerts upon follow-up. Analysis of the warning alerts in the 68 other patients showed that MyPredi^TM^ detected any worsening of the “patient’s health,” with satisfactory results for sensitivity, and negative predictive values (Andrès et al., 2018b).

It should be emphasized that these telemedicine projects are perfectly in line with the “care pathway” theme currently being promoted by the French health authorities (*Ministry of Health and Solidarity*, *Health Insurance*, etc.) in the field of chronic diseases (heart failure, diabetes, COPD, and in the future, long-term COVID and its consequences). These projects undoubtedly contribute to the structuring of patient care pathways. In the field of chronic diseases, and in particular diabetes in the elderly, given the epidemiology of these diseases and the foreseeable shortage of available healthcare time, we need to improve monitoring and education, develop prevention and forecasting, and above all, improve how we select the patients who will need to use the healthcare system.

Nowadays, residents in nursing homes typically have multiple illnesses (cognitive and psycho-behavioral pathologies, undernutrition, heart failure, diabetes, COPD, kidney failure, etc.) and are taking multiple medications. On the medical level, this implies the need for regular monitoring and a high level of medical expertise, including multidisciplinary expertise, for healthcare teams. The GER-e-TEC^TM^ project aims to provide these complex patients with telemedicine tools that enable protocolized and personalized, non-intrusive monitoring. More specifically, the GER-e-TEC^TM^ project takes into account the prevalent issues of aging along with the main geriatric syndromes (falls, malnutrition, cognitive-behavioral disorders, iatropathogenesis, etc.) and does this in the highly specific context of COVID-19-related infection.

With the *MyPredi*^TM^ telemedicine platform, we have demonstrated the value of remote monitoring in the management of elderly patients at the University Hospital of Strasbourg. Moreover, this intelligent platform has been tested in the context of other chronic pathologies through various institutional research projects such as the DIABETe project (Andrès et al., 2018b) (monitoring of patients with type II diabetes). Our work is all the more innovative because remote monitoring was performed on very elderly diabetic patients suffering from COVID-19 infection, which to our knowledge had never been done before, according to recent data in the literature.

As part of the GER-e-TEC project, *MyPredi*^TM^ assists the healthcare staff by automating the processing of information gathered from sensors and questionnaires in order to provide early detection and warning in situations where there is a risk of acute decompensation from a medical point of view. *MyPredi*^TM^ provides a set of recommendations, including operational assistance in emergency situations, and offers accessibility to all healthcare professionals, whether in the home or remotely.

### Limitations

The main weakness of our work lies in the very limited number of elderly patients included for telemonitoring. Indeed, our sample is small (*n* = 10). This is explained by the realization of a study with a monocentric nature, this in an exceptional health environment linked to the COVID-19 epidemic wave.

Our work has other limitations, and we acknowledge that the remote monitoring system put in place can be improved. Indeed, the main limitation of our work lies in the number of alerts issued by our remote monitoring system for the 10 diabetic COVID-19 older patients included. It should be noted that the number of alerts issued for glycemic disorders remains extremely high. Since our remote monitoring solution should be easy to use on a daily basis, it will be necessary to make adjustments to ensure the system does not become overloaded. Smoothing is currently underway, in coordination with the scientific committee steering the study (geriatricians, diabetologists and the Predimed team managing the platform).

## Conclusion

Further investigation of telemonitoring efficacy and cost- effectiveness over longer periods of time, and larger samples is needed.

This work is part of a larger work, consisting in the prevention of geriatric syndromes in COVID-19 older patients: *GER-e-TEC COVID project.*

*GER-e-TEC COVID project* is a unique and innovative project. In fact, to the best of our knowledge, it is the only remote monitoring platform designed to help prevent the deterioration of geriatric syndromes in the era of COVID-19. The main objective of the GER-e-TEC project is the study of the contribution of telemonitoring of dependent elderly patients with a structuring and a protocolization of their medical care, in order to avoid situations of acute decompensation and complications of geriatric risks in COVID-19 patients. The geriatric risks concerned are: falls, constipation, dehydration, confusion, iatrogenia, malnourishment, pain, sleep disorders, heart failure, diabetes, infections, and bedsores.

The results of this 2nd phase of the GER-e-TEC covid study will be communicated later.

## Data Availability Statement

The original contributions presented in the study are included in the article/supplementary material, further inquiries can be directed to the corresponding author/s.

## Ethics Statement

All the COVID-19 older diabetic patients who participated in the GER-e-TEC project were required to sign a consent form. A clinical research protocol for the GER-e-TEC project was filed with the Ethics Committee of the Faculty of Medicine of Strasbourg under the number RNI 2020–HUS N°7792. We also obtained authorization to conduct the study from the Commission Nationale Informatique et Liberté (CNIL, “National Commission on Informatics and Liberty”).

## Author Contributions

A-AZ and EA: conceptualization. A-AZ, MH, and EA: methodology. DM, MH, and AH: software. A-AZ, EA, MH, JH, SE, and AH: validation, writing—review and editing, visualization, project administration, and funding acquisition. A-AZ, DM, MH, and EA: formal analysis. A-AZ: investigation. A-AZ, EA, MH, JH, SE, and AH: resources. A-AZ, DM, MH, and EA: data curation. A-AZ, EA, MH, and AH: writing—original draft preparation. A-AZ, EA, MH, BG, ST, JH, SE, and AH: supervision. All authors have read and agreed to the published version of the manuscript.

## Conflict of Interest

MH is the CEO of predimed. The remaining authors declare that the research was conducted in the absence of any commercial or financial relationships that could be construed as a potential conflict of interest.

## Publisher’s Note

All claims expressed in this article are solely those of the authors and do not necessarily represent those of their affiliated organizations, or those of the publisher, the editors and the reviewers. Any product that may be evaluated in this article, or claim that may be made by its manufacturer, is not guaranteed or endorsed by the publisher.

## Footnotes

1
https://rstudio.com/products/rstudio/download/
